# Maternal peripheral blood platelet‐to‐white blood cell ratio and platelet count as potential diagnostic markers of histological chorioamnionitis‐related spontaneous preterm birth

**DOI:** 10.1002/jcla.22840

**Published:** 2019-02-04

**Authors:** Liyin Qiu, Mian Pan, Ronglian Zhang, Kunhai Ren

**Affiliations:** ^1^ Obstetrical Department, Fujian Provincial Maternity and Children’s Hospital affiliated Hospital of Fujian Medical University Fujian China

**Keywords:** blood platelet‐to‐white blood cell ratio, diagnosis, histological chorioamnionitis, platelet count, preterm birth

## Abstract

**Background:**

Histological chorioamnionitis (HCA) is one of the leading causes of spontaneous preterm birth, thus, to identify novel biomarkers for the early diagnosis of HCA is in a great need.

**Objective:**

To investigate the diagnostic value of maternal peripheral blood platelet‐to‐white blood cell ratio (PLT/WBC) and platelet (PLT) counts in HCA‐related preterm birth.

**Methods:**

A total of 400 patients with preterm birth were enrolled in this study: non‐HCA group (n = 193) and HCA group (n = 207), and 87 full‐term pregnancies were enrolled as the control. The peripheral blood of the participators was collected, and the neutrophil count, WBC count, platelet count, and levels of C‐reactive protein (CRP) and procalcitonin were recorded, and the platelet‐to‐white blood cell ratio (PLT/WBC) of the participators was calculated. Receiver operating characteristic (ROC) curve has been drawn to show the sensitivity and specificity of PLT/WBC and PLT count for the diagnosis of HCA‐related spontaneous preterm birth patients.

**Results:**

The neutrophil count, WBC count, and procalcitonin show no significant differences among the three groups, and the PLT count, PLT/WBC, and CRP (*P* < 0.05) were significantly increased in HCA group compared with non‐HCA group; moreover, the area under the curve (AUC) of PLT/WBC, PLT, and CRP was 0.744 (95% confidence interval [CI], 0.6966‐0.7922), 0.8095 (95% CI, 0.7676‐0.8514), and 0.5730 (95% CI, 0.5173‐0.6287), respectively.

**Conclusion:**

Platelet count and PLT/WBC may become a potential biomarker of HCA‐related spontaneous preterm birth.

## 1 INTRODUCTION

Preterm birth (also named premature birth) is a common obstetrical disorder characterized by premature delivery of the infant at <37 weeks of pregnancy. Based on previous data, the incidence rate of preterm is more than 10% of the total births, and it is also one of the leading causes of pregnancy‐related deaths worldwide (accounts for more than 50% perinatal morbidity and mortality).^1^, ^2^ Preterm birth can be divided into two subtypes, including physician‐initiated birth and spontaneous preterm birth. Spontaneous preterm birth accounts for 2/3 of the total cases of preterm birth, and it has multifactorial etiologies, for example, the premature activation of the fetal endocrine system, intrauterine infection, and pathological distention.^4^, ^5^

In recent years, increasing evidence indicated that there is a strong positive correlation between the infections in the genitourinary tract and the incidence of preterm birth, which consequentially lead to the preterm birth.^6^, ^7^ Chorioamnionitis is one of the most common types of infections in the genitourinary tract during the period of pregnancy, and chorioamnionitis is also one of the leading causes of preterm birth.^6^, ^8^ Results from previous studies indicated that if no proper medical interventions were made at the early stage of chorioamnionitis, premature delivery of the babies from mothers with chorioamnionitis can lead to short‐term and long‐term complications, that is, bronchopulmonary dysplasia (BPD), cerebral palsy (CP), and early‐onset sepsis (EOS).^9^, ^10^ In clinics, chorioamnionitis can be divided into two subtypes: clinical chorioamnionitis and subclinical (or histological chorioamnionitis [HCA]). While clinical chorioamnionitis can be characterized by maternal fever and other symptoms, for example, maternal leukocytosis (>15 000 cells/mm^3^) and maternal tachycardia (>100 bpm), there are no obvious early symptoms for HCA, as a result, to identify novel biomarkers for the early diagnosis of HCA in pregnant women is of great importance for the prevention of spontaneous preterm birth.

In recent years, studies on the serological parameters for the early diagnosis of different diseases have become an area of focus. Some previous studies demonstrated that neutrophil‐to‐lymphocyte ratio of the patients was associated with the diagnosis and outcome of different diseases, including acute cerebrovascular diseases.^11^, ^12^ On the other hand, the neutrophil‐to‐white blood cell ratio (NE/WBC) or platelet‐to‐white blood cell ratio (PLT/WBC) was significantly increased in patients with different type of diseases, for example, cancer, cardiovascular disease, and autoimmune diseases, suggesting that either NE/WBC or PLT/WBC may serve as novel biomarkers for the early diagnosis of those diseases.^15^, ^16^, ^17^

To our knowledge, the roles of PLT/WBC in the pathogenesis of preterm birth have not yet been discussed. In the present study, we will focus on the relationship between PLT/WBC and the chorioamnionitis‐related preterm birth. Our study may provide novel evidence for the application of PLT/WBC as potential biomarkers for the early diagnosis of HCA‐related spontaneous preterm birth.

## 2 METHODS

### 2.1 Patients

A total of 400 patients with preterm birth between November 2016 and April 2017 at Fujian Provincial Maternity and Children’s Hospital, affiliated Hospital of Fujian Medical University, were enrolled in this study. The clinical information of the patients is shown in Table 1. Patients with severe or chronic diseases, including other pregnancy complications (for example, gestational diabetes, pre‐eclampsia, intrahepatic cholestasis of pregnancy, and abruption), cardiovascular disease, autoimmune disease, cancers, diabetes, nephropathy, obesity, and hyperthyroidism, were excluded from this study. Patients were divided into two groups: preterm birth without HCA (n = 197) and preterm birth with HCA (n = 203), and 87 full‐term pregnancies were enrolled as the control group. All participants have signed the written informed consent, and this study has been approved by the Ethics Committee of Fujian Provincial Maternity and Children’s Hospital, affiliated Hospital of Fujian Medical University.

**Table 1:** Clinical characteristics of the participators

Characteristics	Control n = 87	Non‐HCA n = 193	HCA n = 207
Maternal age (y)	28.4 ± 4.2	29.1 ± 4.9	30.7 ± 5.0
Gestational age (wk)	39.8 ± 3.2	32.3 ± 2.8*	32.2 ± 3.6*
Body mass index (kg/m^2^)	20.3 ± 2.0	25.5 ± 3.2*	26.1 ± 3.4*
Birthweight (g)	3428.4 ± 405.7	1880.6 ± 558.8*	1992.8 ± 681.0*

HCA, histological chorioamnionitis.

* *P* < 0.05 vs control.

### 2.2 Diagnosis of HCA

After delivery, tissue samples were obtained from the placenta, umbilical cord (one sample, 2‐4 cm), and placental membranes of the patients. The tissue samples were then embedded into paraffin and sectioned into slices; next, hematoxylin and eosin staining has been performed for the diagnosis of chorioamnionitis. Diagnosis of HCA was determined by the infiltrations of polymorphonuclear leukocytes in the chorion‐decidua as previously described.^18^

### 2.3 Laboratory analysis

Peripheral blood specimens of the patients were collected when they first time come to the outpatient department of our hospital, and none of them received the treatment of corticosteroids, tocolytic agents, or antibiotics. The blood samples of the full‐term pregnancies were collected at the active phase of the first stage of labor. The serological analysis was performed by the clinical laboratory department of Fujian Provincial Maternity and Children’s Hospital, affiliated Hospital of Fujian Medical University. The neutrophil count, WBC count, platelet count, and levels of C‐reactive protein (CRP) and procalcitonin were recorded, and then, platelet‐to‐white blood cell ratio (PLT/WBC) of the participators was calculated.

### 2.4 Statistical analysis

All statistical analyses were performed using the SPSS 19 software package (SPSS Inc, Chicago, IL, USA). Data were presented as mean ± standard deviation (SD). Analysis of variance (ANOVA) has been applied for the comparison of PLT, WBC, NE, and PLT/WBC, has been applied to compare differences among different groups, and Bonferroni correction was used for multiple comparisons. The receiver operating characteristic curve (ROC) has been assessed to determine the diagnostic value of PLT count as well as PLT/WBC *P* < 0.05 was considered as clinical significance.

## 3 RESULTS

### 3.1 Clinical characteristics of the patients

The clinical characteristics of the participators are shown in Table 1. The maternal age has shown no significant differences among the three groups (*P* > 0.05). Compared with the control group, the gestational age and birthweight of the infants were significantly lower in both non‐HCA and HCA groups (*P* < 0.05), and the body mass index in non‐HCA group and HCA group was markedly higher than in the control group (*P* < 0.05).

### 3.2 Elevated PLT count and PLT/WBC in patients with HCA

**Figure 1:**
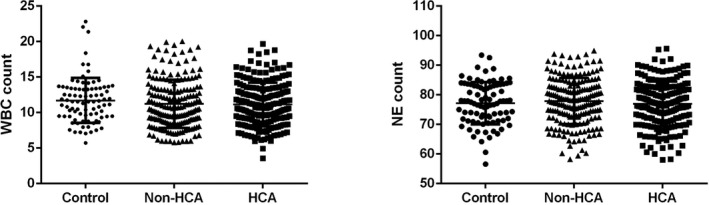
Comparison of the neutrophil count and WBC count among different groups. Control, full‐term pregnancies; HCA, histological chorioamnionitis‐related preterm birth; non‐HCA, non‐histological chorioamnionitis‐related preterm birth; WBC, white blood cell

**Figure 2:**
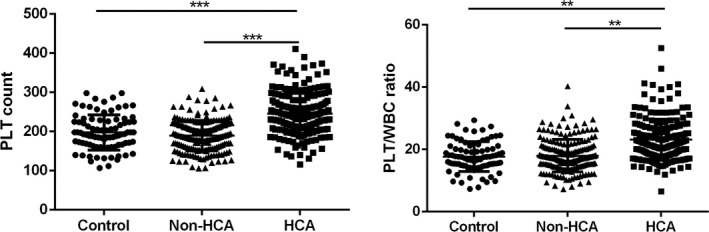
Comparison of the PLT count and PLT/WBC among different groups. Control, full‐term pregnancies; HCA, histological chorioamnionitis‐related preterm birth; non‐HCA, non‐histological chorioamnionitis‐related preterm birth; PLT, platelet; PLT/WBC, platelet‐to‐white blood cell ratio. *P < 0.05, **P < 0.01, ***P < 0.001

Next, we have analyzed the serological data of participators among the three groups. The neutrophil counts and WBC counts show no significant differences among the three groups (Figure 1). The platelet count of the patients was significantly increased in HCA group compared with the control group (*P* < 0.001), and the platelet count in HCA group was significantly higher than in non‐HCA group (*P* < 0.001). On the other hand, the PLT/WBC was significantly increased in HCA group compared with non‐HCA group (*P* < 0.01), and it has shown no significant differences between the control and non‐HCA group (*P* > 0.05; Figure 2).

### 3.3 Comparison of the levels of CRP and procalcitonin in the peripheral blood of the participators

**Figure 3:**
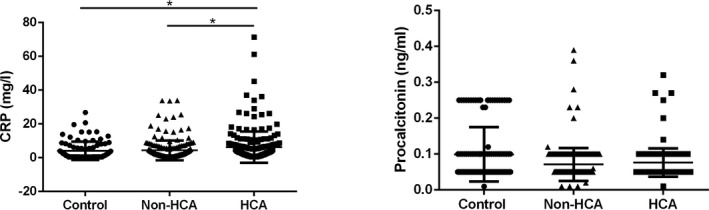
Comparison of the CRP and procalcitonin among different groups. Control, full‐term pregnancies; CRP, C‐reactive protein; HCA, histological chorioamnionitis‐related preterm birth; non‐HCA, non‐histological chorioamnionitis‐related preterm birth. *P < 0.05, **P < 0.01

Moreover, the levels of inflammatory biomarkers CRP and procalcitonin among different groups were compared. As shown in Figure 3, CRP was elevated in HCA group compared with either non‐HCA or the control group (*P* < 0.05); on the other hand, procalcitonin shows no significant difference among the three groups (*P* > 0.05).

### 3.4 PLT/WBC and PLT as early diagnostic markers in patients with HCA

**Figure 4:**
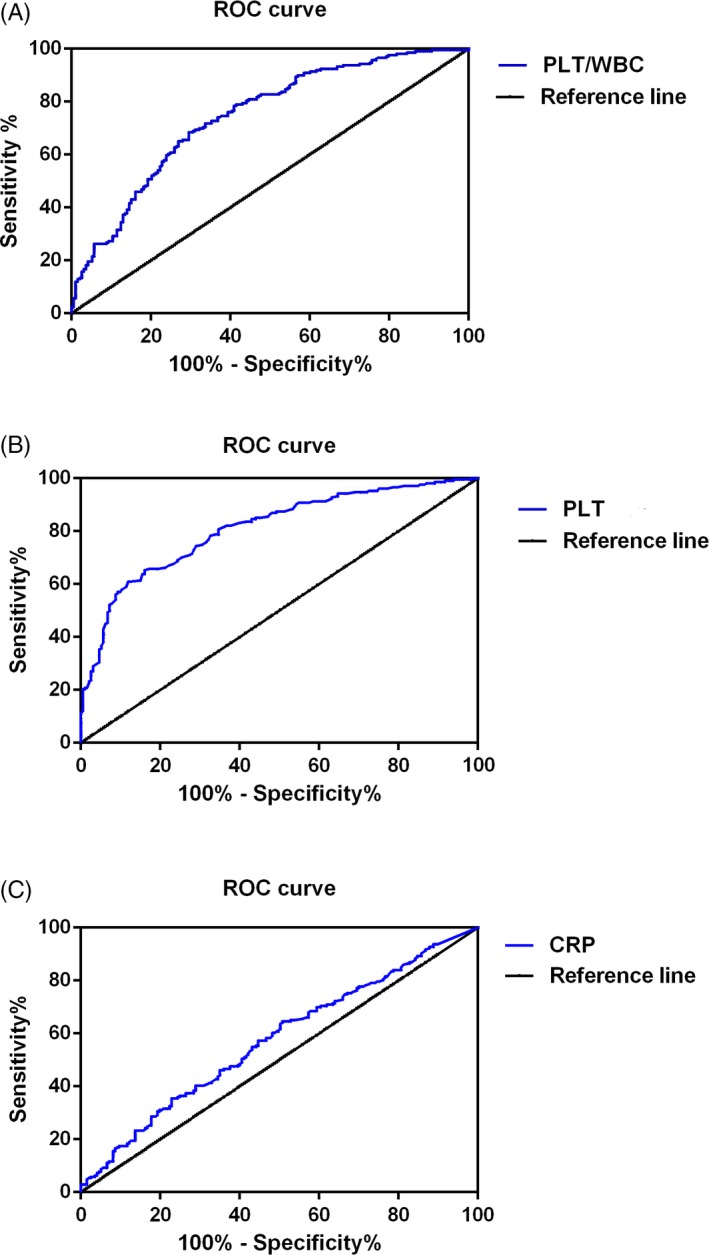
Results of the ROC curve analysis of (A) PLT count, (B) PLT/WBC, and (C) CRP. *P < 0.05, **P < 0.01, ***P < 0.001. CRP, C‐reactive protein; PLT, platelet; PLT/WBC, platelet‐to‐white blood cell ratio; ROC, receiver operating characteristic

Finally, the receiver operating characteristic curves (ROC) have been drawn to show the sensitivity and specificity of PLT/WBC, PLT count, and CRP to distinguish HCA patients from non‐HCA patients. As shown in Figure 4, the area under the curve (AUC) of PLT/WBC was 0.744 (95% confidence interval [CI], 0.6966‐0.7922), and the AUC of PLT was 0.8095 (95% CI, 0.7676‐0.8514), respectively. The AUC of CRP was 0.5730 (95% CI, 0.5173‐0.6287).

## 4 DISCUSSION

In the present study, the roles of PLT/WBC and PLT counts as potential early diagnostic markers for HCA have been discussed. We proved that both PLT/WBC and PLT count were significantly upregulated in patients with HCA, and either PLT/WBC or PLT count was a sensitive biomarker that can distinguish HCA patients from non‐HCA patients with spontaneous preterm birth. Our results have proposed the potential diagnostic value of PLT/WBC and PLT count for the early diagnosis of HCA‐related spontaneous preterm birth.

Intrauterine infection is one of the most common obstetric complications that is induced by chronic or acute inflammation of the membranes and chorion of the placenta, leading to an increased risk of preterm birth. It was estimated that about 40% of cases of preterm birth have been correlated with intrauterine infection.^19^ Infection induced inflammatory condition that involves the recruitment of leukocytes, including neutrophils, monocytes, and lymphocytes; thus, increased number of immune cells in the peripheral blood may indicate the progress of the infectious disease.^16^, ^17^, ^20^, ^21^ In the present study, the neutrophil count and WBC count of control, non‐HCA patients, and HCA patients were compared. To our surprise, the WBC count and neutrophil count showed no significant difference among the three groups (*P* > 0.05). On the other hand, the activation of platelets can be found in the pathophysiological changes in many biological processes, that is, infection, inflammation, and tumorigenesis. In the case of inflammation, platelets were rapidly recruited and participated in the pathogenesis of inflammation by secreting cytokines, chemokines, and other inflammatory mediators^24^, ^25^; however, the relationship between the platelet and HCA has not yet been reported. Interestingly, in our study, we observed that the platelet count of the patients was significantly increased in HCA group compared with the control group (*P* < 0.001), and the platelet count in HCA group was significantly higher than in non‐HCA group (*P* < 0.001); moreover, receiver operating characteristic curve has been drawn to evaluate the diagnostic value of PLT/WBC to distinguish HCA patients from non‐HCA patients. The AUC of PLT count is 0.8095, suggesting that PLT count is a sensitive indicator of HCA among patients with preterm birth. Interestingly, we also observed that CRP, a well‐known inflammatory biomarker, was also elevated in HCA patients, and the AUC of CRP to distinguish HCA patients from non‐HCA patients was 0.5730, indicating that CRP is a not a sensitive biomarker to distinguish HCA patients from non‐HCA patients. Taken together, these results suggested that high maternal peripheral blood level of platelet may indicate the risk of HCA‐related spontaneous preterm birth.

It has been proved in many previous studies that the ratio of immune cells (for example, the NE/WBC ratio, PLT/WBC) in the peripheral blood may serve as inexpensive and reproducible biomarkers. Increasing evidences indicated that peripheral blood PLT/WBC ratio may serve as a biomarker for the early diagnosis, evaluation of the therapeutic efficacy, and prognosis of many diseases.^15^, ^16^, ^17^, ^20^, ^23^ In the present study, the PLT/WBC ratio of different groups was calculated, and we observed that the PLT/WBC was significantly increased in HCA group compared with non‐HCA group (*P* < 0.01); next, results of ROC analysis indicated that the AUC of PLT/WBC was 0.744, suggesting that PLT/WBC is a sensitive biomarker for the diagnosis of HCA. The mechanistic relationship between PLT/WBC ratio and infection remains unclear. One possibility is that the PLT/WBC ratio is an indicator for a patient’s baseline health status. PLT/WBC ratio is also likely an indicator of metabolic syndrome, a constellation of physiological and biochemical abnormalities, resulting in inappropriate activation of inflammatory pathways. Nevertheless, we first reported that PLT/WBC ratio may serve as a biomarker for the early diagnosis of HCA.

Our studies have limitations. First, the results should be verified with larger sample size in future studies; second, this study was based on Chinese Han population, so whether PLT/WBC and PLT have diagnostic value for HCA in other races still needs to be validated.

In conclusion, we reported for the first time that PLT/WBC and PLT count were significantly upregulated in patients with HCA‐related spontaneous preterm birth, and our results have provided novel evidence that PLT/WBC and PLT count were sensitive biomarkers for the early diagnosis of HCA‐related spontaneous preterm birth.
